# Determinants of contraceptive use and future contraceptive intentions of women attending child welfare clinics in urban Ghana

**DOI:** 10.1186/s12889-017-4641-9

**Published:** 2017-08-01

**Authors:** Caroline Wuni, Cornelius A. Turpin, Edward T. Dassah

**Affiliations:** 1 Metropolitan Health Directorate, Kumasi, Ghana; 2 Department of Obstetrics and Gynaecology, Komfo Anokye Teaching Hospital, P. O. Box KS 1934, Kumasi, Ghana; 3 School of Medical Sciences, Kwame Nkrumah University of Science and Technology, Kumasi, Ghana; 4 School of Public Health, Kwame Nkrumah University of Science and Technology, Kumasi, Ghana

**Keywords:** Family planning, Post delivery, Maternal and child health, Child welfare clinic, Current contraceptive use, Future contraceptive intentions, Ghana

## Abstract

**Background:**

Family planning is an integral component of maternal and child health services in Ghana. Although knowledge on contraception is universal and most women attend maternal and child health services, contraceptive use remains low among women after delivery. This study aimed to determine factors influencing current use and future contraceptive intentions of women who were attending child welfare clinics within 2 years of delivery in Sunyani Municipality, Ghana.

**Methods:**

We conducted an analytical cross-sectional study among mothers in six selected health care facilities. Data was collected on their socio-demographic characteristics, reproductive and contraceptive experiences and future contraceptive intentions. Categorical variables were compared using the chi-squared (χ^2^) test. Factors associated with current use and future contraceptive intentions were determined using Poisson regression with a robust error variance to estimate crude and adjusted relative risks (RRs) with 95% confidence intervals (CIs). *P* < 0.1 was considered statistically significant.

**Results:**

A total of 590 women were recruited into the study. Overall, 50.2% of the women were using contraception, 30.7% modern and 19.5% traditional methods. Compared to previous use, more women were using and would prefer the more effective contraceptive methods in future. Significant factors associated with current contraceptive use were, level of education (*p* = 0.02), discussing family planning during antenatal care (adjusted RR, 1.28; 95% CI, 1.07-1.53), or with one’s partner (adjusted RR, 1.22; 95% CI, 1.01-1.47) and previous contraceptive use (adjusted RR, 1.91; 95% CI, 1.56-2.33). Family planning discussions during child welfare clinic (adjusted RR, 1.12; 95% CI, 0.99-1.26) or with one’s spouse (adjusted RR, 1.20; 95% CI, 1.08-1.34), desire to space children (adjusted RR, 1.35; 95% CI, 1.17-1.55), previous (adjusted RR, 1.15; 95% CI, 1.05-1.27) and current (adjusted RR, 1.11; 95% CI, 1.01-1.22) contraceptive use were predictive of clients’ intention to adopt family planning in the future.

**Conclusion:**

Effective counselling on family planning during antenatal and child welfare clinics, and encouraging spousal communication on contraception are likely to increase contraceptive use after delivery.

## Background

Family planning (FP) is critical in accelerating progress towards attainment of the Sustainable Development Goals (SDGs) [1]. FP prevents unwanted pregnancies and associated consequences, maternal and under-5 deaths, and improves the health of both women and children [1]. In most low- and middle-income countries contraceptive use remains low even after delivery, when there is a window of opportunity to improve uptake. Recent estimates from 21 developing countries including Ghana indicate that only 31% of women within the first 2 years of delivery use a FP method [2]. About 77% of postpartum women within this period in Ghana have an unmet need for contraception [2]. Factors that are likely to influence contraceptive use after delivery include, personal, relationship, reproductive and health system factors such as exposure to contraceptive messages during maternal and child health (MCH) care [3, 4]. FP remains an integral component of MCH services in Ghana. Although knowledge on contraception is universal and the majority of women patronise antenatal, postnatal and child health services in Ghana [5], only about one-fifth of women use contraception within 2 years of delivery [2].

MCH services provide opportunities to promote FP due to the frequent encounter of women with the health system and hence increased exposure to FP counselling during these periods [4, 6]. Few studies have examined the determinants of post delivery contraceptive use or intention to use contraception within the context of MCH services in Ghana [6, 7]. In addition to examining other factors, an assessment of FP uptake and future contraceptive intentions during child welfare clinics (CWCs) allows a concomitant evaluation of the effects of exposure to FP messages during the three critical periods of antenatal, postnatal and child health care. Therefore, an analytical cross-sectional study was conducted among women attending CWCs within 2 years of delivery in Sunyani Municipality, Ghana to assess factors influencing contraceptive uptake and their future contraceptive intentions.

## Methods

This analytical cross-sectional study was conducted from 1st to 31st July, 2014 in six selected health care facilities in Sunyani Municipality. All mothers attending CWC at the selected health facilities and whose children were not more than 24 months old within the study period were eligible for inclusion.

Sunyani Municipality is the regional capital, and one of the 27 districts of the Brong Ahafo Region. It is the most urbanized district in the region with 83% of the population living in urban areas and 85% being literate. The municipality has three hospitals, 18 clinics and 46 other health care facilities including community-based health planning and services (CHPS) compounds, maternity homes and health centres. With the exception of the CHPS compounds most of the other facilities provide antenatal and postnatal care, and CWCs also exist in most facilities. FP counselling is an integral component of MCH services in most of the facilities [8].

Six health care facilities, comprising three public (i.e. two hospitals and one health centre) and three private/quasi-government (i.e. one hospital and two maternity homes) facilities offering both child welfare and FP services, and with at least 150 CWC attendees per month, were purposively selected due to sample size considerations. The facilities are; Sunyani Regional Hospital, Sunyani Municipal Hospital, Seventh Day Adventist Hospital, Abessim Health Centre, Monica’s Maternity Home, and Florence Maternity Home.

The participants from each health care facility were systematically selected using the CWC register (records for the day) as the sampling frame. The sampling fraction varied from one in two to one in eight attendees depending on the number of eligible women, with the first person being randomly selected. Selected attendees were approached individually and invited to participate in the study. Consenting clients were interviewed in English or Twi (vernacular) using a pretested structured questionnaire. Data was collected on their socio-demographic characteristics, reproductive and contraceptive experiences as well as future contraceptive intentions.

Sample size calculations were done in Epi Info version 7.1.1.14 (Centers for Disease Control and Prevention; Atlanta, USA). Assuming that the factors influencing contraceptive uptake among women attending CWCs in Sunyani were similar to those observed in Nigeria, level of education, antenatal and postnatal FP counselling, a sample size of 590 had adequate power to detect odds ratios of 1.85, 3.45 and 5.56 respectively [9]. The expected number of clients from each facility was estimated using probability proportional to size.

Data was double entered into Epi Info, cleaned and transferred to Stata Version 12.0 (StataCorp, College Station Texas, USA) for statistical analyses. Categorical variables were compared using the chi-squared (χ^2^) or Fisher’s exact tests as appropriate. To determine the factors associated with current contraceptive use and future contraceptive intentions, crude and adjusted relative risks (RRs) with corresponding 95% confidence intervals (CIs) were calculated using univariable and multivariable Poisson regression with a robust error variance. This regression technique allows an unbiased estimate of the risk when the outcome of interest occurs more than 10% of the time [10], as was the case for most outcomes in this study. Using stepwise backward elimination, factors found to be associated with the outcome of interest at *p* < 0.1 were retained in the multivariable model.

## Results

A total of 590 women who were within 2 years of delivery and attending CWCs in Sunyani Municipality were recruited into the study. The socio-demographic and reproductive characteristics are shown in Table 1. The ages ranged from 13 to 45 years with a mean of 28.9 years (standard deviation 5 years). Over 90% of the mothers were between the ages of 20 and 39 years, with less than 10% being teenagers or in their 40s. Majority (94.2%) of the clients had attained some level of formal education. Most (90.7%) of the respondents were either married or cohabiting with their partners. Of the remaining 9% who were single, 6.6% had never been married while under 3% were separated, divorced or widowed. More than half (58.6%) were employed in the informal sector (farming, trading, sewing) and about a fifth were either unemployed/students, or employed in the formal sector (teaching, nursing, civil service). The median number of living children was 2 (interquartile range 1-3).

**Table 1:** Sociodemographic characteristics of respondents

Variable	Number of women (*N* = 590)	Percentage (%)
Age (completed years)		
13–19	25	4.2
20–29	305	51.7
30–39	251	42.5
40+	9	1.5
Level of education		
No formal education	35	5.9
Basic	285	48.3
Secondary	156	26.4
Tertiary	114	19.3
Marital Status		
Never married	39	6.6
Married	465	78.8
Co-habiting	70	11.9
Divorced/separated/widowed	16	2.7
Occupation		
Student	23	3.9
Unemployed	94	16.0
Informal employment	346	58.6
Formal employment	127	21.5
Number of living children		
1	237	40.2
2	171	30.0
3+	182	30.8

Table 2 shows respondents’ knowledge, past and current use, and future intentions for the various contraceptive methods. Knowledge ranged from 25% for the diaphragm to 98.5% for the male condom. Except for the diaphragm and foam/jelly, knowledge of the shorter acting contraceptives was generally better than that of the long acting reversible contraceptives (implants or intrauterine device [IUD]) or permanent methods (female or male sterilization). Lactational amenorrhoea method (LAM) was the least known traditional method.

**Table 2:** Knowledge, previous and current use, and future intentions for various contraceptive methods

Contraceptive method	Knowledge on method	Last method used	Current method being used	Preferred method in the future
Withdrawal	413 (70.5)	52 (9.0)	24 (4.1)	28 (5.4)
Rhythm	440 (75.1)	111 (18.8)	98 (16.6)	129 (24.8)
Lactational amenorrhea	238 (40.7)	8 (1.4)	28 (4.7)	6 (1.2)
Foam or Jelly	152 (26.0)	2 (0.3)	1 (0.2)	4 (0.8)
Female Condom	442 (75.7)	4 (0.7)	2 (0.3)	7 (1.3)
Male Condom	467 (80.0)	70 (11.9)	33 (5.6)	52 (10.0)
Diaphragm	147 (25.1)	1 (0.2)	4 (0.7)	1 (0.2)
Pill	508 (87.3)	67 (11.4)	43 (7.3)	99 (19.0)
Injectables	498 (85.3)	58 (9.8)	63 (10.7)	148 (28.5)
Implants	394 (67.6)	8 (1.4)	22 (3.7)	40 (7.7)
Intrauterine device (IUD)	330 (56.6)	10 (1.7)	13 (2.2)	23 (4.4)
Female sterilization	329 (56.3)	0 (0)	0 (0)	15 (2.9)
Male sterilization	193 (33.2)	0 (0)	0 (0)	2 (0.4)

Overall, 50.2% of the women were using contraception at the time of the study; 30.7% were using modern contraceptive methods and 19.5% traditional methods. The rhythm method was the most patronised method both prior to, and after the last pregnancy (i.e. during the study period) followed by some shorter acting contraceptives (male condom, injectables and pills). The remaining shorter acting contraceptives (female condom, diaphragm, foam/jelly) were least popular with hardly more than 1% of the women using each method. While less than 2% of the women opted for either of the longer acting reversible contraceptives prior to the index pregnancy for which they were attending the CWC, up to two-and-a-half times that number were using these contraceptives after delivery. As expected, none of the women or their partners had had sterilization prior to the last pregnancy. Into the future, more women would prefer using injectables as compared to the calendar method previously. Generally, there was an increasing trend from prior through current use to future intention to use the more effective reversible contraceptive methods (i.e. IUD and the hormonal methods).

About half (49.3%) of the women obtained their contraceptives from the hospital/FP unit while the remaining half got them from the pharmacies, dispensaries and other outlets.

Common reasons cited for not using contraception after delivery during the study period were; desire to get pregnant at that time (28.6%), previous experience of method-related side effects (28.6%), partner disapproval (9.7%) and religious beliefs (3.7%). About 2% of the women were not using any modern contraceptives at the time because of the cost of the contraceptives.

Univariable and multivariable analyses of factors associated with current contraceptive use are summarised in Table 3. On univariable analysis, significant factors associated current use of contraception after delivery were; level of education, occupation, number of antenatal care (ANC) visits, discussing FP during ANC or with one’s partner, desire to space children, resuming sexual intercourse and previous contraceptive use. On multivariable analysis, level of education (*p* = 0.02), discussing FP during ANC (adjusted RR, 1.28; 95% CI, 1.07-1.53), discussing FP with one’s partner (adjusted RR, 1.22; 95% CI, 1.01-1.47) and previous contraceptive use (adjusted RR, 1.91; 95% CI, 1.56-2.33) remained significantly associated with current contraceptive use. Current contraceptive use increased with increasing level of education (p trend = 0.03).

**Table 3:** Factors associated with current contraceptive use among women attending child welfare clinic (CWC) in Sunyani Municipal, Ghana

Variable	Current contraceptive use n (%)	Unadjusted RR (95% CI)	Adjusted RR (95% CI)
Age (completed years)		*p* = 0.45	
13–19	9 (36.0)	1	−
20–29	154 (50.8)	1.41 (0.83, 2.41)	−
30+	131 (50.6)	1.40 (0.82, 2.40)	−
Level of education		*p* = 0.001; p trend < 0.001	*p* = 0.02; p trend = 0.03
No formal education	16 (38.1)	1	1
≤ Basic education	123 (44.6)	1.17 (0.78, 1.76)	0.90 (0.64, 1.25)
> Basic education	154 (59.0)	1.55 (1.04, 2.31)	1.13 (0.82, 1.58)
Marital Status		*p* = 0.33	
Single	24 (43.6)	1	−
Married	270 (50.0)	1.17 (0.86, 1.60)	−
Occupation		*p* = 0.01	*p* = 0.11
Unemployed	46 (39.6)	1	1
Semi-skilled employment	172 (50.4)	1.27 (0.99, 1.63)	1.29 (1.01, 1.66)
Skilled employment	75 (59.5)	1.50 (1.15, 1.96)	1.20 (0.92, 1.56)
Number of living children		*p* = 0.32	
1–2	196 (48.6)	1	−
3+	97 (53.0)	1.09 (0.92, 1.29)	−
Number of ANC visits		*p* = 0.06	*p* = 0.24
< 7	109 (46.2)	1	1
7+	184 (54.3)	1.18 (0.99, 1.39)	1.10 (0.94, 1.29)
FP discussed during ANC		*p* = 0.01	*p* = 0.001
No	71 (41.8)	1	1
Yes	208 (58.9)	1.41 (1.16, 1.72)	1.28 (1.07, 1.53)
FP discussed during PNC		*p* = 0.33	
No	12 (46.0)	1	−
Yes	283 (51.5)	1.24 (0.80, 1.93)	−
FP discussed during CWC		*p* = 0.56	
No	17 (51.4)	1	−
Yes	271 (51.1)	1.11 (0.78, 1.59)	−
Discusses FP with partner		*p* < 0.001	*p* = 0.03
No	74 (37.0)	1	1
Yes	217 (60.6)	1.64 (1.34, 2.00)	1.22 (1.01, 1.47)
Desire to space children		*p* < 0.001	*p* = 0.14
No	54 (36.2)	1	1
Yes	242 (54.9)	1.51 (1.20, 1.90)	1.16 (0.95, 1.41)
Resumed sexual intercourse		*p* = 0.01	*p* = 0.70
No	192 (46.8)	1	1
Yes	104 (57.8)	1.23 (1.05, 1.45)	1.03 (0.89, 1.19)
Resumed menses		*p* = 0.61	
No	215 (49.5)	1.05 (0.88, 1.25)	−
Yes	81 (52.9)	1	−
Previous contraceptive use		*p* < 0.001	*p* < 0.001
No	82 (28.5)	1	1
Yes	214 (70.9)	2.45 (2.04, 3.03)	1.91 (1.56, 2.33)

*RR* relative risk, *CI* confidence interval, *ANC* antenatal care, *FP* family planning, *PNC* postnatal care

On future contraceptive intentions, discussing FP during CWC (adjusted RR, 1.12; 95% CI, 0.99-1.26), client discussing FP with her partner (adjusted RR, 1.20; 95% CI, 1.08-1.34), desire to space children (adjusted RR, 1.35; 95% CI, 1.17-1.55), previous contraceptive use (adjusted RR, 1.15; 95% CI, 1.05-1.27) and current contraceptive use (adjusted RR, 1.11; 95% CI, 1.01-1.22) were predictive of clients’ intention to use contraception in the future (Table 4). On the whole, clients discussing FP with their partners and previous contraceptive use were significant determinants of both current use and future intentions to use contraception.

**Table 4:** Factors associated with mothers’ intention to use contraceptives in the future among women attending child welfare clinic (CWC) in Sunyani Municipal, Ghana

Variable	Intention to use contraceptives in the future	Unadjusted RR (95% CI)	Adjusted RR (95% CI)
Age (completed years)		*p* = 0.15	
13–19	22 (88.0)	1.17 (1.00, 1.37)	−
20–29	230 (75.9)	1.01 (0.92, 1.11)	−
30+	195 (75.3)	1	−
Level of education		*p* = 0.18	
No formal education	26 (61.9)	1	−
≤ Basic education	215 (77.9)	1.26 (0.98, 1.61)	−
> Basic education	202 (77.4)	1.25 (0.98, 1.60)	−
Marital Status		*p* = 0.68	
Single	43 (78.2)	1.03 (0.89, 1.20)	−
Married	401 (75.8)	1	−
Occupation		*p* = 0.77	
Unemployed	87 (75.0)	1.01 (0.87, 1.16)	−
Semi-skilled employment	264 (77.4)	1.04 (0.92, 1.17)	−
Skilled employment	94 (74.6)	1	−
Number of living children		*p* = 0.25	
1–2	300 (74.4)	1	−
3+	144 (78.7)	1.06 (0.96, 1.16)	−
Number of ANC visits		*p* = 0.29	
< 7	185 (78.4)	1.05 (0.96, 1.15)	−
7+	253 (74.6)	1	−
FP discussed during ANC		*p* = 0.18	
No	125 (73.5)	1	−
Yes	279 (79.0)	1.07 (0.97, 1.19)	−
FP discussed during PNC		*p* = 0.66	
No	21 (72.4)	1	−
Yes	410 (76.2)	1.05 (0.84, 1.32)	−
FP discussed during CWC		*p* = 0.04	*p* = 0.08
No	32 (86.5)	1.15 (1.00, 1.32)	1.12 (0.99, 1.26)
Yes	398 (75.1)	1	1
Discusses FP with partner		*p* < 0.001	*p* = 0.001
No	130 (65.0)	1	1
Yes	308 (86.0)	1.32 (1.19, 1.48)	1.20 (1.08, 1.34)
Desire to space children		*p* < 0.001	*p* < 0.001
No	86 (57.7)	1	1
Yes	362 (82.1)	1.42 (1.23, 1.64)	1.35 (1.17, 1.55)
Previous contraceptive use		*p* < 0.001	*p* = 0.004
No	187 (64.9)	1	1
Yes	261 (86.4)	1.33 (1.21, 1.47)	1.15 (1.05, 1.27)
Current contraceptive use		*p* < 0.001	*p* = 0.02
No	179 (66.1)	1	1
Yes	259 (85.8)	1.30 (1.18, 1.43)	1.11 (1.01, 1.22)

*RR* relative risk, *CI* confidence interval, *ANC* antenatal care, *FP* family planning, *PNC* postnatal care, *CWC* child welfare clinic

## Discussion

The prevalence of contraceptive use among women attending CWCs in Sunyani Municipal is quite impressive. Educational background and discussing FP during ANC were associated with current contraceptive use, while discussing FP during CWC, desire to space children and current contraceptive use were significant determinants of clients’ intention to use contraception in the future. Spousal communication on FP and previous contraceptive use were predictive of both current uptake and intention to adopt FP in the future.

The contraceptive prevalence of 50% among women attending CWC in Sunyani Municipality within 24 months of delivery is much higher than the rate of 27% among currently married women reported in the 2014 Ghana Demographic and Health Survey (GDHS) [5], and 39% of postpartum women within 2-12 months of delivery who had been referred to KATH [7]. The higher contraceptive use in the current study could be attributed to the higher proportion of women in their peak reproductive ages of 20-39 years (94% compared to 63%) and four-fold uptake of traditional methods (20% vs. 5%) in the current study compared to women in the GDHS. In the KATH study, contraceptive uptake was assessed within 12 months of delivery, when postpartum amenorrhoea, abstinence and insusceptibility to pregnancy occur and the women are less likely to use contraception [3, 5]. The current study included women who were outside this period of insusceptibility to pregnancy, and hence were more likely to use contraception.

Similar to findings of the 2014 GDHS [5], knowledge on contraceptive methods was almost universal. While a good number of the clients relied on traditional methods, it is gratifying to note that use of more effective contraceptive methods (hormonal methods, IUD and sterilization) increased after the current delivery compared to previous use of contraceptives. Although the use of long-acting reversible contraceptives was generally low, increases of up to two-and-a-half times were observed in use of these methods. These findings suggest that the women were now relying on more effective methods. This trend could also translate into their future contraceptive uptake, as higher proportions of women expressed their desire to adopt more effective methods in the future. The use of highly effective contraceptive methods will ensure adequate child spacing and prevent the unwanted consequences of short inter-pregnancy interval and unintended pregnancies.

Consistent with the results of previous studies [4, 11–13], we found that level of education, discussing FP during ANC or with one’s partner and previous use of contraceptives were significantly associated with current use of contraception. The findings on women’s educational level underscore the importance of education on FP uptake, as less educated women were less likely to use contraceptives. Therefore, it is imperative that FP service providers must place special emphasis on, and address the needs of women of lower educational status during FP sessions to improve their contraceptive use [4, 13]. The results also highlight the need to take advantage of the antenatal period as a window of opportunity for promoting contraceptive use after delivery. Providing comprehensive ANC including effective counselling on contraception does not only improve the quality of care during pregnancy and client satisfaction, but has the potential to increase contraceptive use after childbirth [4, 11]. Although FP counselling is supposed to be an integral component of postnatal care and CWC, such discussions are often overlooked or inadequate in content, as priority is given to addressing complications associated with delivery, and the welfare and immunization of the baby [11, 14]. Consequently, contraceptive counselling after delivery may not adequately promote FP [11, 14], as was observed in this study.

There is some evidence that health care workers could be trained/retrained to provide more effective FP services during CWC through group health education sessions, distribution of simple educational material on postpartum FP, individualized counselling and initiation of chosen contraceptive method [15–17]. Group health education talks during these clinics should discuss the risk of becoming pregnant, benefits of FP as well safe and effective contraceptive options within this period. Simple brochures on postpartum FP, which should be suitable for the local context, could also be provided to the mothers so that they could share the information with their spouses at home. One-on-one interactive counselling session with each mother at CWC (e.g. while she is waiting for, or as the baby receives immunization) will help identify women who require special attention or more information (such as women with low educational level). The woman’s contraceptive method of choice can be initiated after these educational and counselling sessions [15–17]. Mothers’ knowledge and satisfaction with these interventions can be assessed for purposes of improved service delivery. These interventions often require adequate stocks of contraceptives and infrastructure including space for individual counselling at CWC, and regular monitoring and supervision [15, 16]. The cooperation of relevant stakeholders in FP in the country [15, 16], both governmental (Ministry of Health/Ghana Health Service) and non-governmental organisations including donors and service delivery groups (e.g. World Health Organization, United States Agency for International Development [USAID], United Kingdom Department for International Development, Planned Parenthood Association of Ghana, Marie Stopes International) is required to provide the necessary support and training in this regard. Generally, these interventions have been found to cost-effective, improve contraceptive uptake after delivery and strengthen MCH services [15–17]. However, in one study in Ghana and Zambia, incomplete implementation of these interventions did not result in a significant increase in contraceptive uptake [18].

In agreement with the findings of other studies, spousal communication on contraception was found to increase uptake. Improved partner discussion on FP enhances knowledge, understanding and consensus on contraceptive issues between couples with resultant increase in contraceptive practice [7, 12, 19]. In one study in Northern Ghana, spousal discussion on FP was a predictor of contraceptive uptake as well as women’s intention to adopt FP in the future [12]. At the national level, nearly two-thirds (63%) of married women using contraception in Ghana jointly take those decisions with their partners [5].

The factors associated with women’s intention to practice FP in the future in this study, have been reported previously [6, 20]. Women’s desire to space births, previous and current contraceptive use were predictive of their intention to use FP in the future. Since contraceptive counselling during CWC and spousal discussion on FP both influenced women’s intention to adopt contraception in the future, there is need to encourage effective contraceptive counselling after delivery and involve partners during FP sessions. It is pertinent to note that future contraceptive intentions do not necessarily translate into uptake. More often than not, fewer women than the number who intended to use contraception, actually adopt a FP method [6].

The study had some limitations. Smaller health care facilities were excluded mainly due to sample size considerations. The conduct of MCH services including FP counselling and contraceptive practices among CWC attendees in these smaller facilities may be different from those of the larger facilities. Women who do not seek MCH services were also excluded. As such findings from this study cannot be generalised to other health care facilities or women who do attend MCH clinics. Second, our main outcome, contraceptive use, is sensitive information, which was self-reported and could be subject to reporting bias. Third, it is possible that women who elected to use contraception before pregnancy were more likely to recall having received counselling during ANC or with their partners, thereby weakening the association between contraceptive use and the counselling received.

## Conclusion

The prevalence of contraceptive use among women attending CWC in Sunyani Municipality was impressive. Compared to previous use, more women were using and would prefer the more effective FP methods in the future. Significant factors influencing current use and future contraceptive intentions related to client socio-demographic characteristics, reproductive wishes and contraceptive experiences, spousal communication and FP counselling during ANC and CWC. Therefore, effective contraceptive counselling during ANC and CWC, and encouraging spousal discussions on FP are likely to increase contraceptive uptake after childbirth. Strategies to improve contraceptive counselling during postnatal care and CWC, and male involvement in FP should be explored.

## Abbreviations

ANC: antenatal care
CHPS: community-based health planning and services
CI: confidence interval
CWC: child welfare clinic
FP: family planning
GDHS: Ghana Demographic and Health Survey
IUD: intrauterine device
KATH: Komfo Anokye Teaching Hospital
LAM: lactational amenorrhoea method
MCH: maternal and child health
RR: relative risk
SDGs: Sustainable Development Goals
